# Traumatic pneumorrhachis associated with cerebral fluid leakage evaluated with magnetic resonance myelography

**DOI:** 10.1002/ccr3.7200

**Published:** 2023-05-30

**Authors:** Kazuki Sugaya, Ken Iseki

**Affiliations:** ^1^ Department of Emergency Medicine Fukushima Medical University School of Medicine Fukushima Japan

**Keywords:** cerebrospinal fluid leakage, magnetic resonance myelography, Pneumoencephalopathy, Pneumorrhachis, trauma

## Abstract

Attention should be paid to cerebrospinal fluid leakage in patients with pneumorrhachis associated with vertebral body trauma. If pneumorrhachis is detected, further imaging investigation and bed rest should be considered as appropriate.

## 1 INTRODUCTION

An 88‐year‐old male patient suffered from a fall injury and was transported to our emergency center. Body CT showed a fracture at the 11th thoracic vertebra and air within the intradural spinal canal. Magnetic resonance myelography was useful to evaluate cerebrospinal fluid leakage. Traumatic pneumorrhachis with vertebral body fracture should be considered during cerebral fluid leakage.

Air contained within the spinal canal is termed as pneumorrhachis. It is a rare condition, although its rate of discovery has been increasing with recent developments in imaging techniques. In general, the distribution of emphysema to the subarachnoid or epidural layer is clinically important. In cases of subarachnoid emphysema, attention should be paid to the onset of cerebral hernia and meningitis associated with cerebrospinal fluid leakage. In the present report, magnetic resonance (MR) myelography was used to evaluate the presence of cerebrospinal fluid leakage in a case of vertebral body fractures caused by falling from a height of at least 3 m.

## 2 CASE PRESENTATION

An 88‐year‐old male patient with a history of hypertension and chronic obstructive pulmonary disease, who suffered a fall from a height of 3 m followed by a blow to the buttocks, was brought to our department. Observation by the ambulance crew revealed that his vital signs were stable, and he was able to walk with assistance.

When he arrived at our hospital, his consciousness level was Glasgow Coma Scale E4V5M6 (eyes opened spontaneously, verbal response was orientated, best motor response was obeyed commands); body temperature, 37.3°C; blood pressure, 134/83 mmHg; heart rate, 73 beats/min; respiratory rate, 20 breaths/min; and oxygen saturation (SpO_2_), 97% at rest in room temperature air. He complained of lumbar and back pain, and physical examination revealed bruises on his buttocks. However, there were no other symptoms or neurological abnormalities, such as limb numbness, paralysis, or bladder‐rectal disturbances. He had no otorrhea or rhinorrhea suggestive of cerebrospinal fluid leakage.

Laboratory evaluation revealed mild normocytic anemia (red blood cells 4.14 × 10^6^/μl, hemoglobin 13.0 g/dL, mean corpuscular volume 92.8 fl), thrombocytopenia (platelet 15.7 × 10^4^/μl), and elevated D‐dimer level (D‐dimer 68.5 μg/mL), which was speculated to be caused by trauma. Since there was no progression of thrombocytopenia and no bleeding tendency, the patient was followed up. A head computed tomography (CT) showed free air in the left inferior horn of the left ventricle, the frontal longitudinal fissure, and the base of the skull. The patient had no skull base fracture or traumatic intracranial hemorrhage. Chest CT showed an 11th thoracic vertebral fracture. He also had air in his thoracic spinal canal and a thoracic epidural hematoma from the 4th to 8th thoracic vertebrae. He had posterior pneumomediastinum but no pneumothorax, and the continuity of the esophageal mucosa was preserved. He also had bilateral minor hemothorax and fractures of the right 10th and 11th ribs.

The patient was hospitalized and treated conservatively for these injuries. Because he had pneumorrhachis with pneumoencephalopathy, we determined that emphysema was distributed in the subarachnoid space. However, since the portal of entry was unknown, and the possibility of cerebrospinal fluid leakage could not be ruled out, he was confined to bed rest in a supine position at 0°. Posterior pneumomediastinum was also observed, and the possibility of an esophageal injury was considered. However, esophagography could not be performed because the head could not be lifted owing to the patient's movement restriction. Although there was concern about the onset of meningitis and mediastinitis, prophylactic antibiotics were not administered for the early detection of the disease.

**FIGURE 1:**
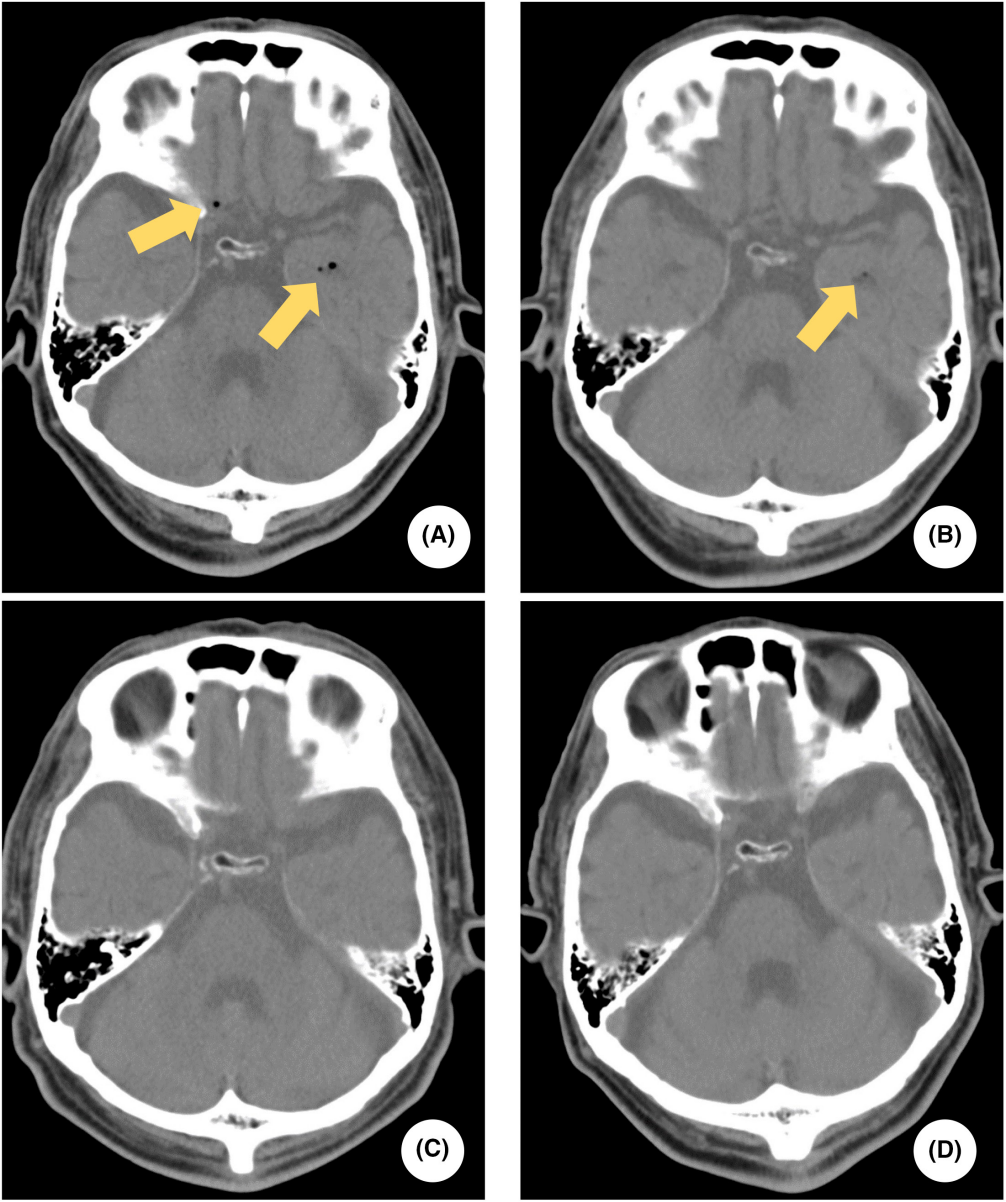
Head computed tomography (CT) of the patient. Pneumocephalus (yellow arrows) is shown in A and B of the head CT image. A：Day 1, B：Day 2, C：Day 4, D：Day 9.

**FIGURE 2:**
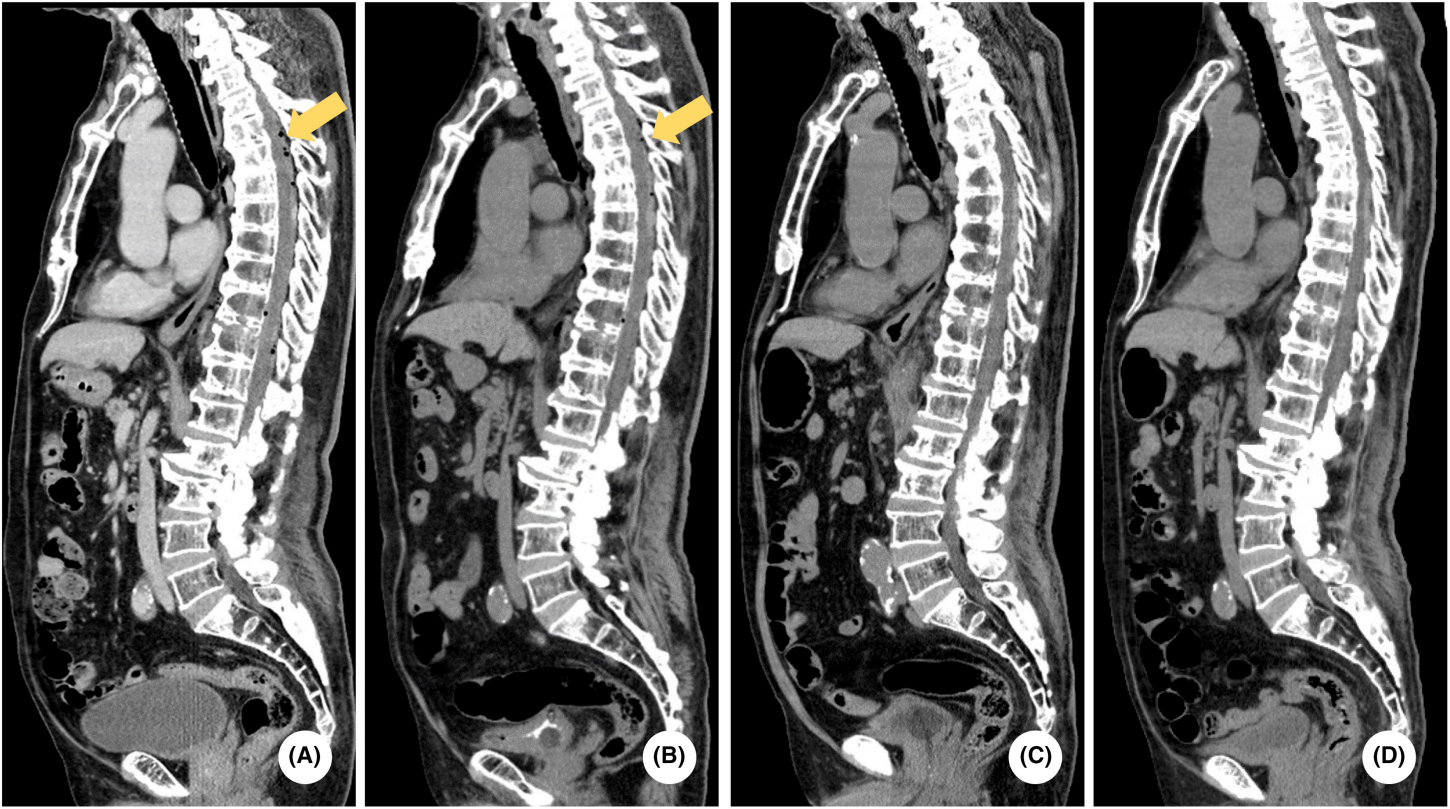
Computed tomography (CT) of the patient’s spine. Pneumorrhachis (yellow arrows) is shown in A and B of the spinal CT image. A：Day 1, B：Day 2, C：Day 4, D：Day 9.

**FIGURE 3:**
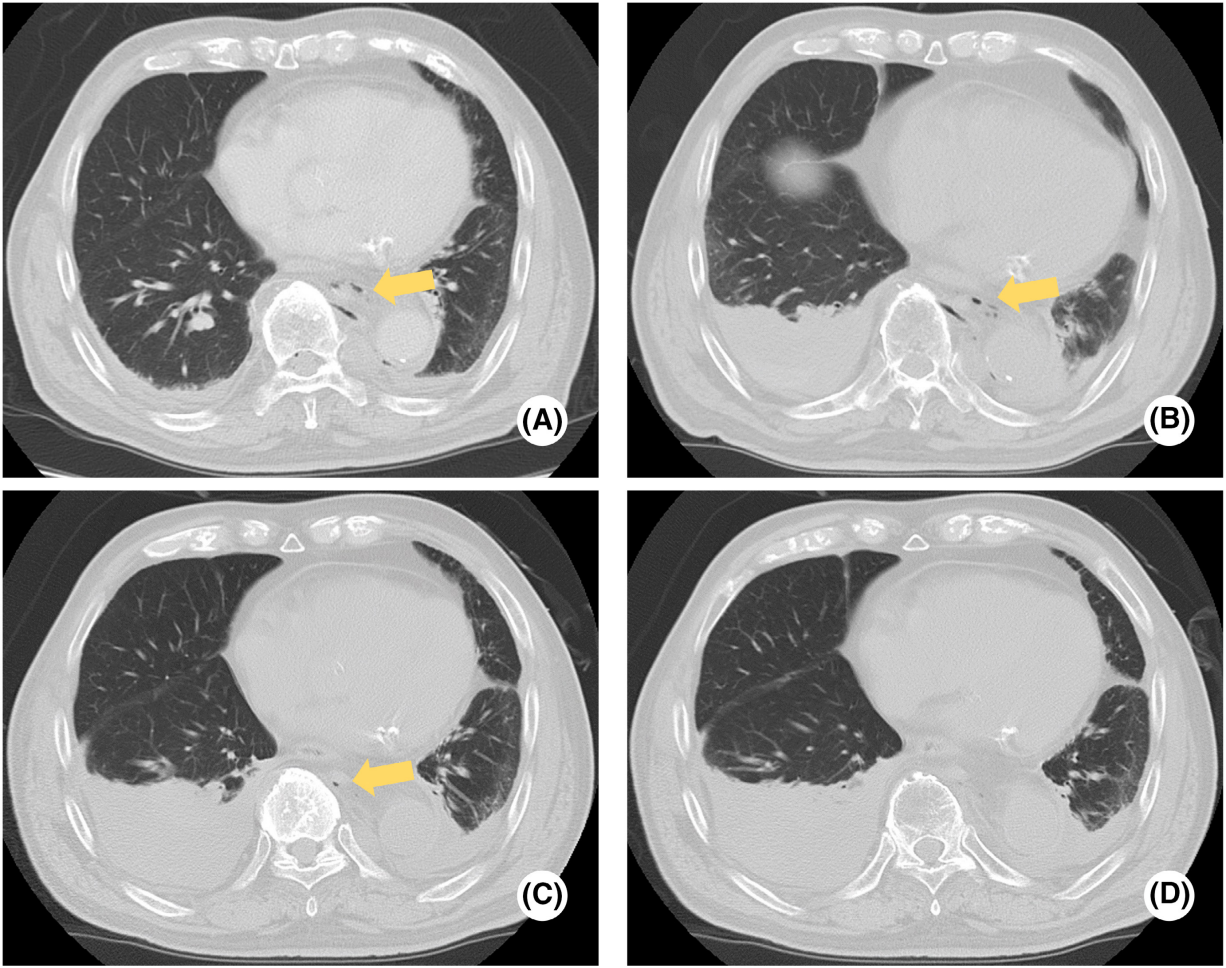
Chest computed tomography (CT) of the patient. Posterior pneumomediastinum (yellow arrows) shown in A, B, and C of the chest CT image. A：Day 1, B：Day 2, C：Day 4, D：Day 9.

**FIGURE 4:**
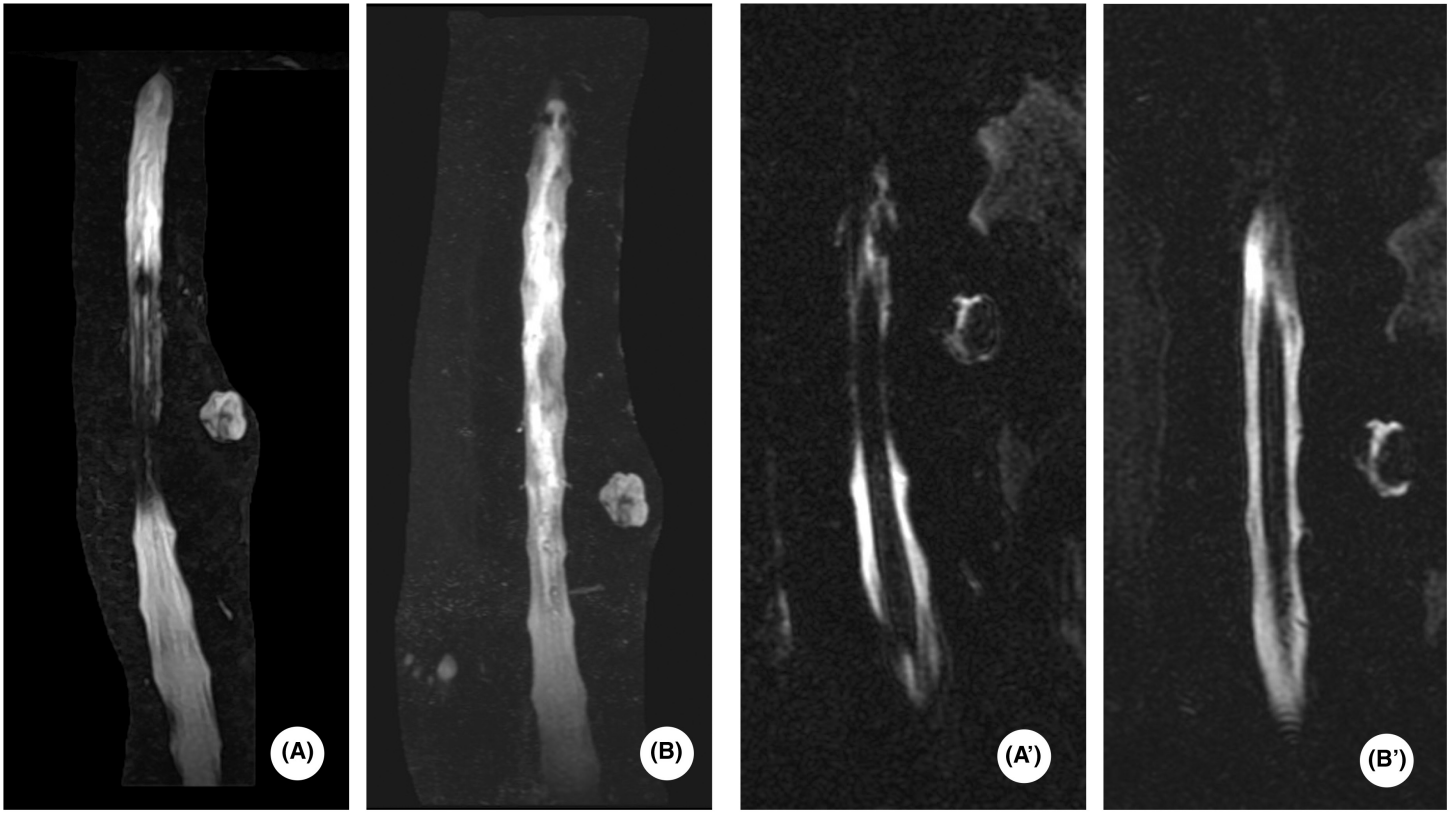
Magnetic resonance (MR) myelography images are shown in A and B. Spinal MR cholangiopancreatography (MRCP) is shown in (A′, B′).

**FIGURE 5:**
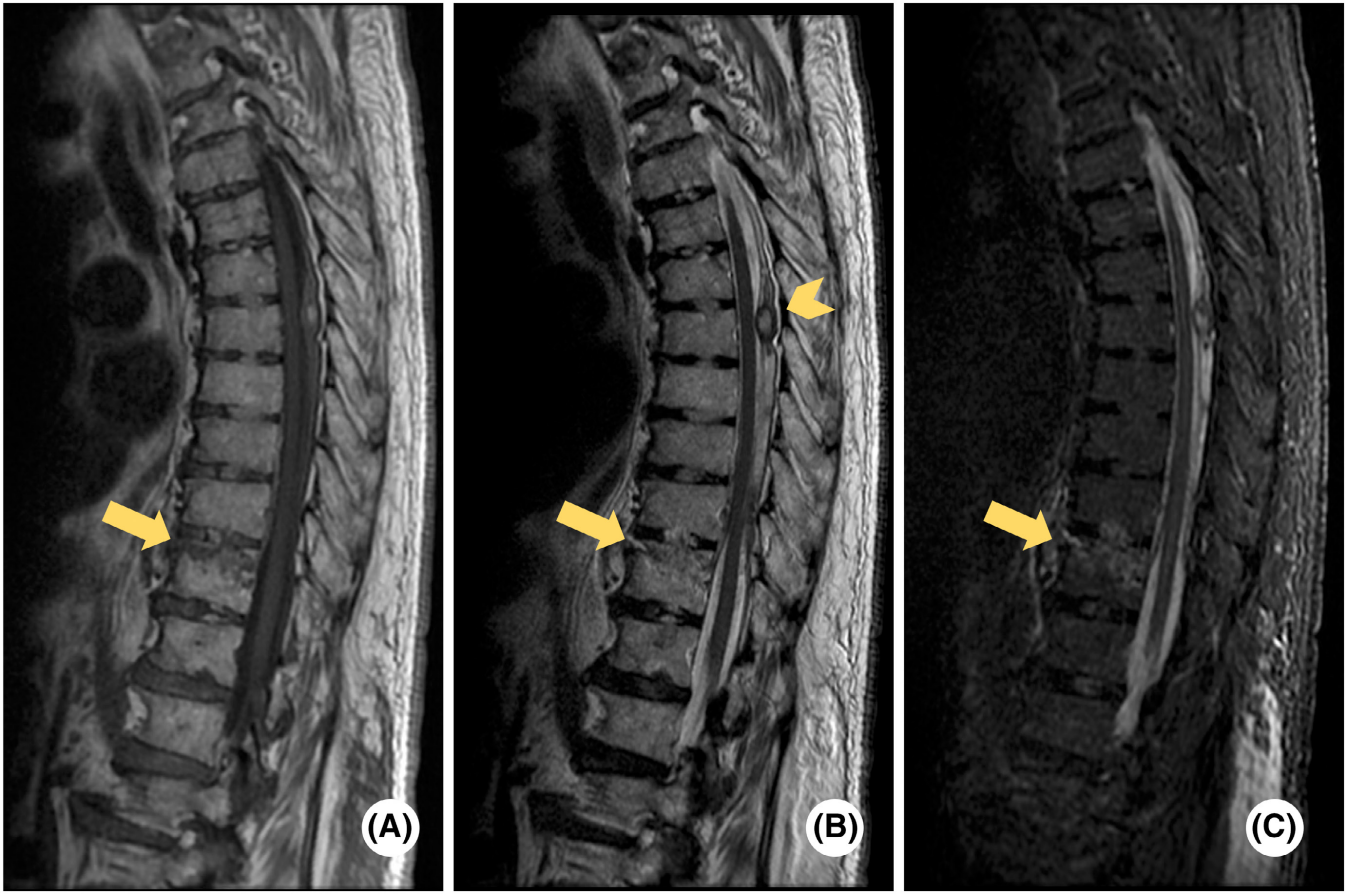
Spinal magnetic resonance imaging (MRI) on Day 3. Thoracic vertebral fracture (yellow arrows) shown in (A–C) of spinal MRI. Thoracic epidural hematoma indicated by yellow arrowhead in B.

**FIGURE 6:**
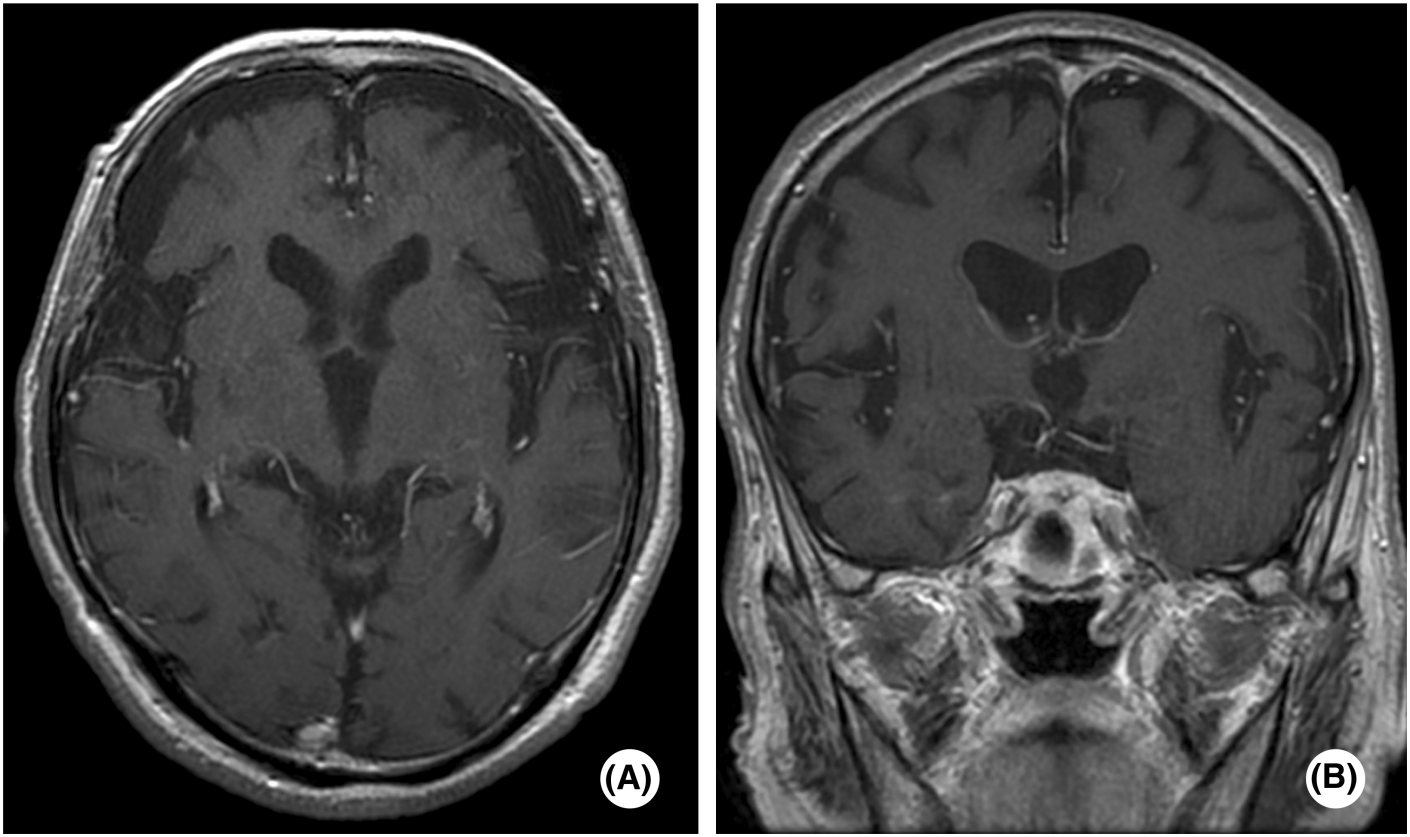
Contrast‐enhanced magnetic resonance imaging (MRI) of head computed tomography (CT).

Findings of the whole‐body CT were reexamined on Day 2 (Figures 1 and 2). Exacerbation of bilateral dorsal atelectasis and hemothorax associated with bed rest was observed (Figure 3). On the same day, oral intake of nutritional supplements was initiated, while paying attention to the physical findings of mediastinitis. On Day 3, vertebral MR imaging (MRI; 1.5 Tesla, GE Healthcare, MR450W1.5) was performed to detect cerebrospinal fluid leakage and assess vertebral fractures. Myelography and magnetic resonance cholangiopancreatography (MRCP) did not show clear extra cerebrospinal fluid hyperintensity, and the possibility of cerebrospinal fluid leakage was judged to be low (Figure 4). Additionally, the 11th thoracic vertebra showed a high signal in short TI inversion recovery (STIR), confirming that it was a new thoracic vertebral fracture (Figure 5). In consultation with the department of neurosurgery, the patient was allowed to raise his head up to an angle of 20°in accordance with the degree of rest suggested for thoracic vertebral fractures. On Day 4, whole‐body CT was performed again, and pneumoencephalopathy and pneumorrhachis were not observed. Posterior mediastinal emphysema remained present, but was insignificant. As the patient's respiratory status was stable, he was followed up without chest drainage. On Day 9, whole‐body CT was repeated to evaluate the lung fields, and the bilateral dorsal atelectasis and hemothorax were unchanged. His posterior pneumomediastinum had disappeared, and pneumoencephalopathy and pneumorrhachis also remained absent. On Day 10, the use of corset was considered no longer essential as the patient lifted his head with the corset on, but there were no signs of cerebrospinal fluid leakage. On Day 15, MR myelography was performed again, but no extra cerebrospinal fluid was detected. On Day 16, a cranial contrast‐enhanced MRI was performed; however, no dural thickening suggestive of cerebrospinal fluid hypovolemia was detected (Figure 6).

The patient was transferred to a neighboring hospital on Day 19 for continuous rehabilitation without any notable sequelae.

## 3 DISCUSSION

Herein, we report a case of vertebral body fracture due to a fall, complicated by pneumorrhachis, subarachnoid emphysema, posterior mediastinum, and thoracic epidural hematoma.

In 1977, Gordon and Hardman first reported pneumorrhachis in the cervical spine of a patient who had suffered a base skull fracture in a motor vehicle accident.^1^ They hypothesized that the head‐down positioning used by emergency medical personnel during resuscitation allowed air to migrate caudally to the cervical subarachnoid space. Pneumorrhachis of the thoracolumbar spine was first described by Prins and Vencken in 1989.^2^ A 40‐year‐old man who fell down a flight of stairs presented with a skull base fracture, leukoencephalopathy, and pneumorrhachis in the lumbar spinal canal. Similar to Gordon and Hardman's report, the authors reported that placing the head in a low position during resuscitation moved the air caudally. Both cases are thought to have been caused by air contamination associated with base skull fractures; however, in the present case, base skull fractures were not observed, and the portal of entry was unknown. Pneumorrhachis is usually diagnosed incidentally and represents an asymptomatic epiphenomenon of coincident underlying injuries and diseases. To the best of our knowledge, only 48 cases of traumatic pneumorrhachis have been reported up to 2022.

Pneumorrhachis diagnosis is done by radiography, CT, and MRI, and it is almost impossible to diagnose clinical symptoms.^3^ X‐rays are the least sensitive method and can only detect large amounts of air in the spinal canal.^4^ Pneumorrhachis is generally asymptomatic; however, when associated with basilar skull fractures and cerebrospinal fluid leakage, it may require intervention. In other words, whether emphysema's distribution site is the subarachnoid space or epidural space is clinically important; however, even with CT, it is difficult to distinguish between them. According to Gelalis et al., emphysema in the subarachnoid space is associated with more severe trauma as compared to emphysema in the epidural space, which is considered harmless.^4^ In the present case, no clinical findings suggestive of pneumorrhachis were observed. Regarding the distribution of emphysema, we speculated that emphysema was distributed in the subarachnoid space because it was accompanied by pneumoencephalopathy. However, their distribution was similar to that of an epidural hematoma; as mentioned above, it was difficult to distinguish.

The etiologies of pneumomorrhachis are classified as iatrogenic, nontraumatic, and traumatic.^4^ Iatrogenic pneumorrhachis is caused by surgical and anesthetic procedures.^5^, ^6^, ^7^ Nontraumatic pneumorrhachis is caused by malignant tumors, severe cough due to bronchial asthma or bronchitis, resuscitation treatment for cardiopulmonary arrest due to suffocation, post‐exercise, inhalation drug abuse, and frequent vomiting due to diabetic ketoacidosis.^8^, ^9^, ^10^, ^11^, ^12^, ^13^ Traumatic pneumorrhachis is associated with various injuries, including head injuries. Pneumorrhachis rarely occurs independently, and most cases are associated with trauma. Two hypotheses have been proposed for the mechanism of air entrainment into the spinal canal; however, the mechanism has not yet been clarified. In other words, there is no fascia between the posterior mediastinal space and the dural space; therefore, air generated by pneumothorax or pneumomediastinum enters the nerve roots through the neural foramen due to pressure.^4^ Another potential pathway for pneumorrhachis is air embolization of the mediastinum veins and vertebral vein plexus from the air localized in the mediastinum.^4^ In the present case, posterior pneumomediastinum was observed; therefore, the intrusion of air into the spinal canal is thought to have originated in the mediastinum, but the mechanism by which the posterior pneumomediastinum occurs is unknown. Due to the present patient's history of chronic obstructive pulmonary disease, the possibility of minor alveolar or airway damage was considered, but pneumothorax was not evident on CT upon admission. In addition, although the possibility of esophageal damage has not been proven, as described above, the onset of mediastinitis is considered negative because there was no onset of mediastinitis even after eating.

In the present case, leukoencephalopathy was observed; therefore, it was judged that air existed in the subarachnoid space, and cerebrospinal fluid leakage was evaluated using MR myelography. In general, when cerebrospinal fluid leakage is suspected, head MRI, MR myelography, intrathecal gadolinium injection MR myelography, and CT myelography are considered.^14^ For intrathecal gadolinium injection MR myelography, lumbar puncture could not be performed because of bed rest restrictions. In addition, MR myelography has a non‐inferior diagnostic rate compared with intrathecal gadolinium‐ injection MR myelography.^15^ We had planned to proceed with early mobilization if there was no cerebrospinal fluid leakage, but this was not possible because of the restriction of the patient's movements due to the vertebral body fracture. MR myelography is non‐invasive, easy to use, and can be used to evaluate vertebral body fractures; therefore, if pneumorrhachis associated with vertebral trauma is diagnosed, MR myelography should be considered.

Conservative treatment is the first choice of treatment for cerebrospinal fluid leakage.^14^ In such situations, the case typically resolves after a few weeks of bed rest. However, epidural blood patch therapy is selected when relief cannot be achieved with conservative treatment.^16^ As the patient in the current case report also had thoracic epidural hematoma, it was speculated that the hematoma played the role of a blood patch, and spontaneous remission was obtained without clinical symptoms of cerebrospinal fluid leakage.

## 4 CONCLUSION

Attention should be paid to cerebrospinal fluid leakage in patients with pneumorrhachis associated with vertebral body trauma. If pneumorrhachis is detected, imaging studies and bed rest should be considered appropriate. Pneumorrhachis can be relieved with conservative treatment, and it has been suggested that epidural hematoma may play the role of a blood patch.

## AUTHOR CONTRIBUTIONS

**Kazuki Sugaya:** Writing – original draft; writing – review and editing. **Ken Iseki:** Supervision.

## FUNDING INFORMATION

This research received no specific grants from any funding agency in the public, commercial, or not‐for‐profit sectors.

## CONFLICT OF INTEREST STATEMENT

All the authors declare no competing interests.

## ETHICAL APPROVAL

This study was performed in accordance with the ethical standards of the Japan Association for Acute Medicine Society.

## CONSENT

Written informed consent was obtained from the patient for publication of this case report and accompanying dates and images.

## Data Availability

The data that support the findings of this study are available from the corresponding author upon reasonable request.
